# Supplementary material for: Things we can do now that we could not do before: Developing and using a cross-scalar, state-wide database to support geomorphologically-informed river management
Source: PLoS One. 2021 Jan 22;16(1):e0244719. doi: 10.1371/journal.pone.0244719 (PMC7822514; doi:10.1371/journal.pone.0244719)
Supplement: S1 Table — Note: the raw data in the master database has been processed to produce this summary. (DOCX) [file pone.0244719.s001.docx]

**S1 Table** Total stream length of River Styles in the NSW River Styles database. Note: the raw data in the master database has been processed to produce this summary.

| **River Style** | **Statewide total stream length (km)** | **Statewide (%)** | **North Coast (%)** | **South Coast (%)** | **Sydney Metro (%)** | **Inland (%)** |
| --- | --- | --- | --- | --- | --- | --- |
| **Confined** | **72107.36** | **33.3** | **59.3** | **52.5** | **74.8** | **18.4** |
| CVS - Gorge | 19636.7 | 9.1 | 17.2 | 23.4 | 48.3 | 3.4 |
| CVS - Headwater | 23596.6 | 10.9 | 20.0 | 12.7 | 26.5 | 6.2 |
| CVS - Occasional floodplain pockets, gravel | 20480.6 | 9.5 | 15.7 | 11.6 |  | 6.2 |
| CVS - Occasional floodplain pockets, sand | 8393.5 | 3.9 | 6.4 | 4.8 |  | 2.5 |
| **Partly confined** | **48902.39** | **22.6** | **25.9** | **20.9** |  | **21.2** |
| PCVS - Bedrock controlled, fine grained | 2870.8 | 1.3 | 1.4 | 0.8 |  | 1.4 |
| PCVS - Bedrock controlled, gravel | 11928.1 | 5.5 | 5.1 | 6.2 |  | 5.6 |
| PCVS - Bedrock controlled, low sinuosity, gravel | 0.2 | <0.1 | <0.1 |  |  |  |
| PCVS - Bedrock controlled, sand | 4321.5 | 2.0 | 1.0 | 3.2 |  | 2.4 |
| PCVS - Dune controlled | 6.3 | <0.1 | <0.1 |  |  |  |
| PCVS - Dune controlled, anabranching | 178.0 | <0.1 |  |  |  | 0.1 |
| PCVS - Planform controlled, anabranching | 117.6 | <0.1 |  |  |  | <0.1 |
| PCVS - Planform controlled, low sinuosity, cobble | 148.2 | <0.1 | 0.1 |  |  | <0.1 |
| PCVS - Planform controlled, low sinuosity, fine grained | 5100.6 | 2.4 | 1.5 | 2.9 |  | 2.7 |
| PCVS - Planform controlled, low sinuosity, gravel | 11466.3 | 5.3 | 8.5 | 5.2 |  | 3.8 |
| PCVS - Planform controlled, low sinuosity, sand | 7704.9 | 3.6 | 3.2 | 2.7 |  | 3.9 |
| PCVS - Planform controlled, meandering, fine grained | 1972.2 | 0.9 | 1.9 |  |  | 0.5 |
| PCVS - Planform controlled, meandering, gravel | 1714.2 | 0.8 | 1.7 |  |  | 0.4 |
| PCVS - Planform controlled, meandering, sand | 1172.9 | 0.5 | 1.6 |  |  | <0.1 |
| PCVS - Planform controlled, wandering, sand | 200.5 | <0.1 |  |  |  | 0.1 |
| **Laterally unconfined – continuous channel** | **63042.06** | **29.1** | **8.0** | **14.8** | **24.8** | **40.9** |
| LUV CC - Anabranching | 6043.3 | 2.8 |  |  |  | 4.5 |
| LUV CC - Anabranching, gravel | 441.8 | 0.2 |  |  |  | 0.3 |
| LUV CC - Anabranching, swamp belt | 137.5 | <0.1 | 0.2 |  |  |  |
| LUV CC - Anastomosing | 320.0 | 0.1 |  |  |  | 0.2 |
| LUV CC - Bank confined, fine grained | 1276.1 | 0.6 | 0.3 |  |  | 0.8 |
| LUV CC - Bank confined, gravel | 56.1 | <0.1 | <0.1 |  |  | <0.1 |
| LUV CC - Bank confined, sand | 1097.3 | 0.5 | <0.1 |  |  | 0.8 |
| LUV CC - Channelised fill | 12464.4 | 5.8 | 2.9 | 8.6 | 2.2 | 6.8 |
| LUV CC - Low sinuosity, entrenched gravel | 33.7 | <0.1 | <0.1 | 0.0 |  |  |
| LUV CC - Low sinuosity, fine grained | 15596.7 | 7.2 | 1.7 | 2.2 | 21.5 | 10.4 |
| LUV CC - Low sinuosity, gravel | 3913.8 | 1.8 | 0.2 | 2.2 |  | 2.5 |
| LUV CC - Low sinuosity, multi-channel sand belt | 2.1 | <0.1 | <0.1 |  |  |  |
| LUV CC - Low sinuosity, sand | 3920.1 | 1.8 | 0.4 | 1.1 |  | 2.6 |
| LUV CC - Meandering, entrenched gravel | 97.8 | <0.1 | 0.1 | 0.0 |  |  |
| LUV CC - Meandering, fine grained | 16006.1 | 7.4 | 1.1 | 0.5 | 1.0 | 11.2 |
| LUV CC - Meandering, gravel | 532.8 | 0.2 | 0.2 | 0.2 | 0.2 | 0.3 |
| LUV CC - Meandering, sand | 646.1 | 0.3 | 0.6 |  | <0.1 | 0.2 |
| LUV CC - Multi-channel, sand belt | 1.5 | <0.1 | <0.1 | 0.0 |  |  |
| LUV CC - Wandering, gravel | 334.1 | 0.2 |  | 0.0 |  | 0.2 |
| LUV CC - Wandering, sand | 120.5 | <0.1 | <0.1 | 0.0 |  | <0.1 |
| **Laterally unconfined – discontinuous channel** | **32565.62** | **15.0** | **6.8** | **11.7** | **0.4** | **19.5** |
| LUV DC - Chain of ponds | 2197.4 | 1.0 | 0.6 | 1.0 |  | 1.2 |
| LUV DC - Cut and fill | 6082.2 | 2.8 | 2.2 |  |  | 3.4 |
| LUV DC - Discontinuous sand bed | 5.9 | <0.1 | <0.1 |  |  |  |
| LUV DC - Dune controlled, chain of ponds | 752.1 | 0.3 |  |  |  | 0.6 |
| LUV DC - Dune controlled, floodout | 417.7 | 0.2 |  |  |  | 0.3 |
| LUV DC - Floodout | 4308.5 | 2.0 | <0.1 |  | 0.4 | 3.2 |
| LUV DC - Valley fill, fine grained | 14990.5 | 6.9 | 3.0 | 9.3 |  | 8.6 |
| LUV DC - Valley fill, sand | 3811.4 | 1.8 | 0.9 | 1.4 |  | 2.2 |
