# Supplementary material for: Things we can do now that we could not do before: Developing and using a cross-scalar, state-wide database to support geomorphologically-informed river management
Source: PLoS One. 2021 Jan 22;16(1):e0244719. doi: 10.1371/journal.pone.0244719 (PMC7822514; doi:10.1371/journal.pone.0244719)
Supplement: S2 Table — Scores in bold are below average. (DOCX) [file pone.0244719.s002.docx]

**S2** **Table** Scores for RSGCI by catchment. Scores in **bold** are below average.

| **Catchment** | **RSGCI** |
| --- | --- |
| **North Coast** | |
| Central Coast | 0.69 |
| Hawkesbury-Nepean | 0.82 |
| Hunter | **0.37** |
| Lower North Coast | 0.66 |
| Northern Rivers | 0.65 |
| **South Coast** | |
| Southern Rivers | 0.72 |
| **Sydney Metro** | |
| Sydney Metro | 0.77 |
| **Inland** | |
| Barwon Darling | 0.75 |
| Border Rivers | **0.58** |
| Central West | **0.59** |
| Gwydir | **0.54** |
| Lachlan | 0.63 |
| Lower Murray-Darling | **0.61** |
| Murray | **0.51** |
| Murrumbidgee | **0.52** |
| Namoi | **0.40** |
| Western | 0.76 |
