# Supplementary material for: Things we can do now that we could not do before: Developing and using a cross-scalar, state-wide database to support geomorphologically-informed river management
Source: PLoS One. 2021 Jan 22;16(1):e0244719. doi: 10.1371/journal.pone.0244719 (PMC7822514; doi:10.1371/journal.pone.0244719)
Supplement: S3 Table — Note: the raw data in the master database has been processed to produce this summary. (DOCX) [file pone.0244719.s003.docx]

**S3 Table** Percentages of stream length in each region and catchment that are in different recovery potential and prioritisation classes in the NSW River Styles database. Note: the raw data in the master database has been processed to produce this summary.

|  | **Conservation (%)** | **Strategic (%)** | **High recovery potential (%)** | **Moderate recovery potential (%)** | **Low recovery potential (%)** | **No data (%)** |
| --- | --- | --- | --- | --- | --- | --- |
| **North Coast** | **44.5** | **2.6** | **17.5** | **28.4** | **6.6** | **0.4** |
| Central Coast | 42.4 | 13.5 | 34.5 | 8.2 | 0.7 | 0.6 |
| Hawkesbury-Nepean | 68.6 | 2.9 | 9.0 | 17.3 | 2.1 | 0.2 |
| Hunter | 19.4 | 4.5 | 20.4 | 38.5 | 16.9 | 0.3 |
| Lower North Coast | 47.7 | 1.4 | 26.4 | 20.7 | 1.7 | 2.2 |
| Northern Rivers | 43.9 | 1.4 | 17.1 | 32.5 | 5.0 | 0.0 |
| **South Coast** | **55.5** | **2.1** | **10.7** | **24.8** | **6.9** |  |
| Southern Rivers | 55.5 | 2.1 | 10.7 | 24.8 | 6.9 | 0.0 |
| **Sydney Metro** | **61.6** |  | **23.8** | **10.6** | **4.0** |  |
| Sydney Metro | 61.6 | 0.0 | 23.8 | 10.6 | 4.0 | 0.0 |
| **Inland** | **34.5** | **5.3** | **18.5** | **23.0** | **18.5** | **0.2** |
| Barwon Darling | 49.3 | 0.0 | 5.8 | 44.9 | 0.0 | 0.0 |
| Border Rivers | 34.9 | 14.5 | 13.8 | 20.4 | 16.5 | 0.0 |
| Central West | 38.5 | 14.6 | 2.8 | 11.5 | 32.5 | 0.1 |
| Gwydir | 25.3 | 16.4 | 21.1 | 23.7 | 13.5 | 0.0 |
| Lachlan | 43.6 | 1.2 | 18.9 | 19.0 | 17.2 | 0.1 |
| Lower Murray-Darling | 37.7 | 0.2 | 19.4 | 29.9 | 12.7 | 0.0 |
| Murray | 12.0 | 6.8 | 17.5 | 51.9 | 11.7 | 0.0 |
| Murrumbidgee | 26.6 | 0.3 | 11.2 | 44.7 | 17.1 | 0.0 |
| Namoi | 17.1 | 4.2 | 29.7 | 24.9 | 23.5 | 0.6 |
| Western | 61.3 | 0.3 | 29.7 | 0.0 | 8.7 | 0.0 |
